# P-117. Clinical and Epidemiological Characteristics of Balamuthia mandrillaris Infection in Peru: A Retrospective Case Series

**DOI:** 10.1093/ofid/ofaf695.345

**Published:** 2026-01-11

**Authors:** Eduardo Gotuzzo, Dalila Martinez, Blas M Cornejo-Esparza, Daniel Guillen, Carlos Seas, Francisco G Bravo

**Affiliations:** Instituto de Medicina Tropical Alexander von Humboldt, Universidad Peruana Cayetano Heredia, Lima, Lima, Peru; Hospital Nacional Cayetano Heredia, Lima, Lima, Peru; Instituto de Medicina Tropical Alexander von Humboldt - Universidad Peruana Cayetano Heredia, Lima, Lima, Peru; Hospital Nacional Cayetano Heredia, Lima, Lima, Peru; Instituto de Medicina Tropical Alexander von Humboldt - UPCH, Lima, Lima, Peru; Universidad Peruana Cayetano Heredia, Lima, Lima, Peru

## Abstract

**Background:**

*Balamuthia mandrillaris* is a neglected free-living ameba causing cutaneous lesions and granulomatous encephalitis with high mortality. Diagnostic testing is limited, and cases may be underdiagnosed and underreported. A low threshold of clinical suspicion should prompt workup and treatment. Miltefosine was introduced in the treatment regimen in 2006. We aim to describe the epidemiological, clinical, and anatomopathological characteristics of *B. mandrillaris* infection in the Peruvian population.

**Table 1.**
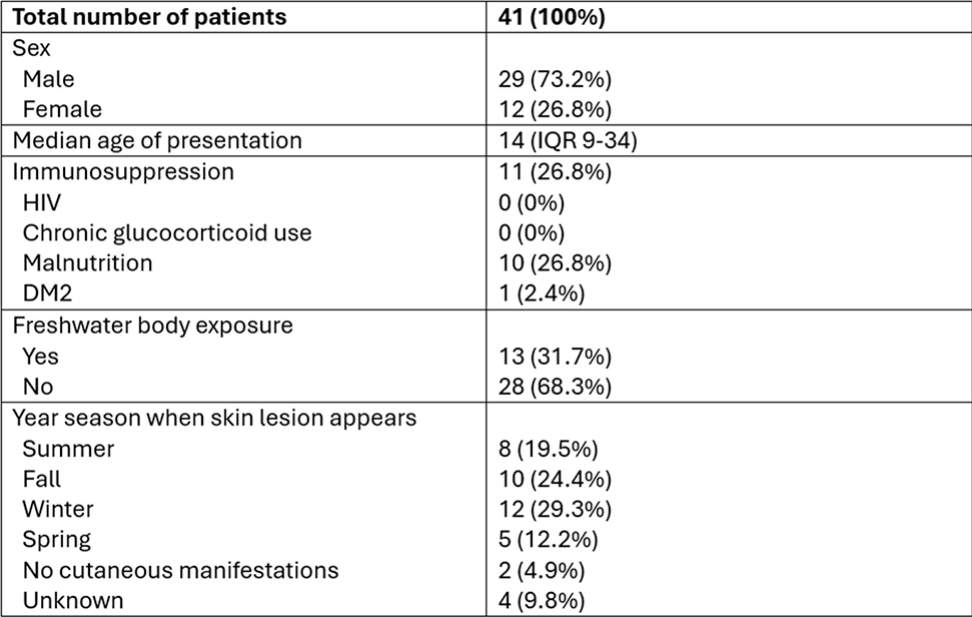
Patient Characteristics

**Table 2.**
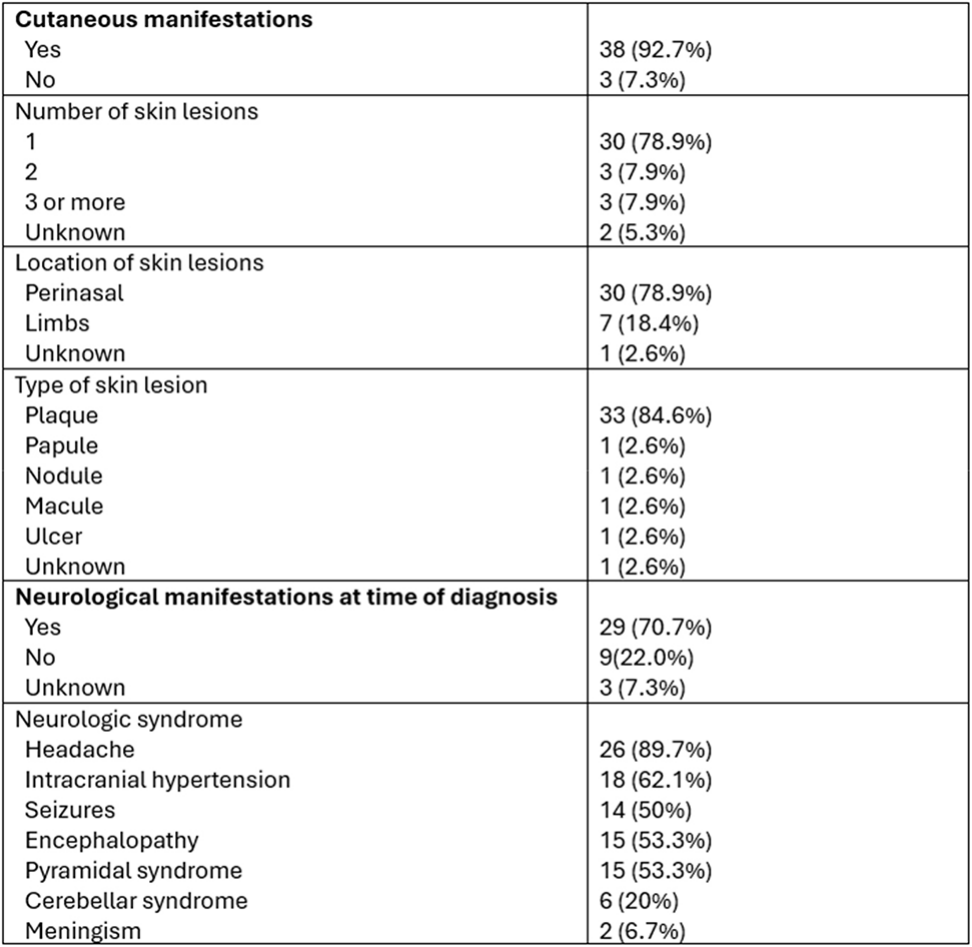
Clinical Characteristics

**Methods:**

We performed a retrospective review of medical records of patients with *B. mandrillaris* infection presenting to Hospital Cayetano Heredia from January 1975 to December 2017. Disease confirmation was defined as positive immunofluorescence testing for *B. mandrillaris* of brain or skin biopsies or direct visualization of *B. mandrillaris* trophozoites on biopsy of patients with a compatible clinical syndrome. Analysis was performed using Stata software.

**Figure 1.**
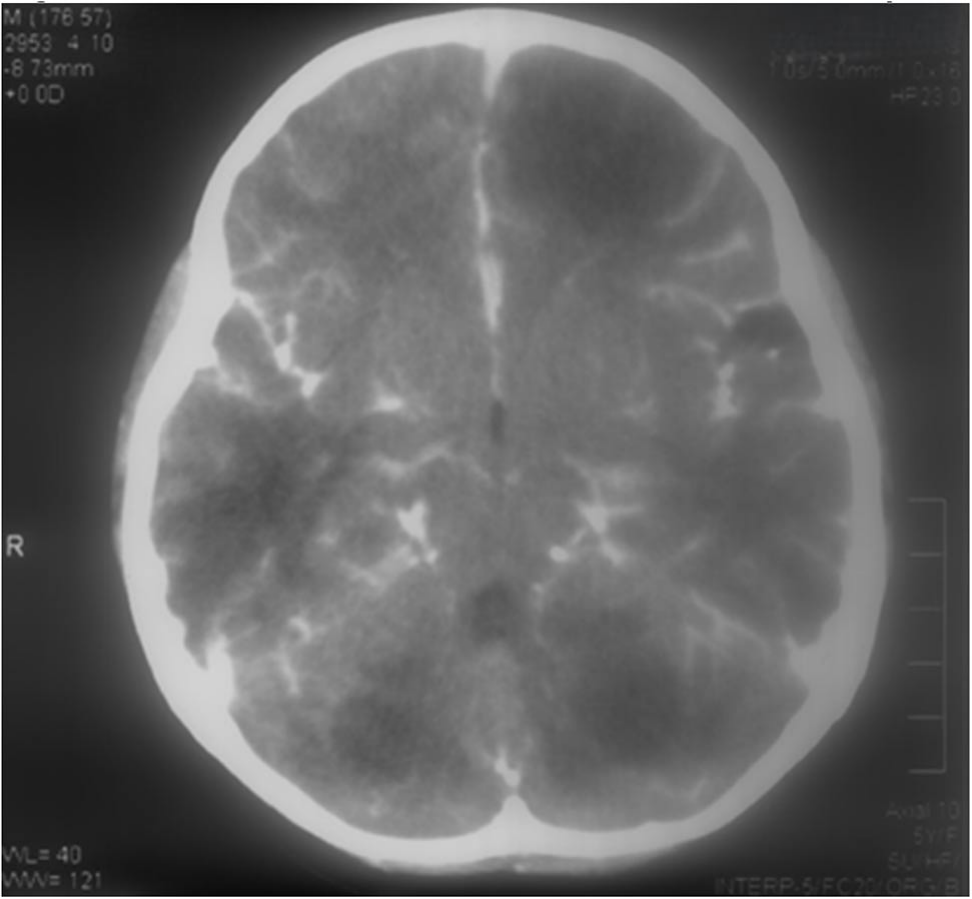
Contrast Brain CT Scan of B. mandrillaris encephalitis patient

**Figure 2.**
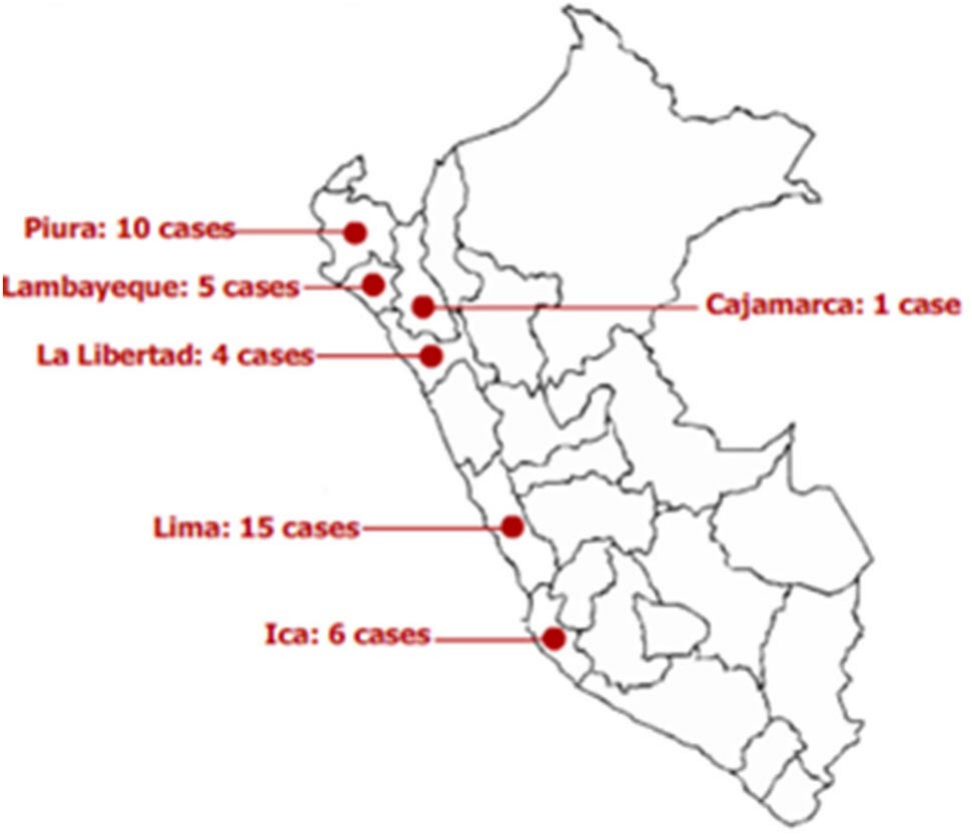
Patient place of origin

**Results:**

Sixty-eight cases of suspected *B. mandrillaris* infection were identified: 41 have positive immunofluorescence testing or direct trophozoite identification on biopsy, and 27 are still under investigation. Patient characteristics of the 41 confirmed cases are summarized in Table 1. No patients had HIV infection; 24 (58.5%) died, 9 (22.0%) were lost to follow-up, and 8 (19.5%) patients survived. Morality was 91.7% in patients with neurologic manifestations. The most common type of skin lesion was a centro-facial indurated, painless, erythematous plaque. Median time from cutaneous involvement until onset of neurologic symptoms was 169.5 days (IQR 56-389). Median time from neurologic symptom onset until death was 38 days (IQR 22-87.5).

**Conclusion:**

Most patients with *B. mandrillaris* encephalitis died of the disease. All confirmed cases came from the coast of Peru, most presented during winter or fall. No patients had HIV or other causes of immunosuppression besides malnutrition. Early recognition and prompt initiation of appropriate treatment before developing neurologic manifestations may improve survival.

**Disclosures:**

All Authors: No reported disclosures

